# Reconciling Autonomy and Beneficence in Treatment Decision-Making for Companion Animal Patients

**DOI:** 10.1007/s10991-018-9211-4

**Published:** 2018-06-02

**Authors:** Carol Gray, Marie Fox, Pru Hobson-West

**Affiliations:** 1 School of Law, University of Birmingham, Birmingham, UK; 2 School of Law and Social Justice, University of Liverpool, Liverpool, UK; 3 Centre for Applied Bioethics, School of Veterinary Medicine and Science, University of Nottingham, Nottingham, UK

**Keywords:** Informed consent, Veterinary medicine, Autonomy, Beneficence

## Abstract

This article explores how the concept of consent to medical treatment applies in the veterinary context, and aims to evaluate normative justifications for owner consent to treatment of animal patients. We trace the evolution of the test for valid consent in human health decision-making, against a backdrop of increased recognition of the importance of patient rights and a gradual judicial espousal of a doctrine of informed consent grounded in a particular understanding of autonomy. We argue that, notwithstanding the adoption of a similar discourse of informed consent in professional veterinary codes, notions of autonomy and informed consent are not easily transferrable to the veterinary medicine context, given inter alia the tripartite relationship between veterinary professional, owner and animal patient. We suggest that a more appropriate, albeit inexact, analogy may be drawn with paediatric practice which is premised on a similarly tripartite relationship and where decisions must be reached in the best interests of the child. However, acknowledging the legal status of animals as property and how consent to veterinary treatment is predicated on the animal owner’s willingness and ability to pay, we propose that the appropriate response is for veterinary professionals generally to accept the client’s choice, provided this is informed. Yet such client autonomy must be limited where animal welfare concerns exist, so that beneficence continues to play an important role in the veterinary context. We suggest that this ‘middle road’ should be reflected in professional veterinary guidance.

## Introduction

Veterinary surgeons must communicate effectively with clients and ensure informed consent is obtained before treatments or procedures are carried out. (Royal College of Veterinary Surgeons 2017a)

In its Code of Professional Conduct, the Royal College of Veterinary Surgeons (RCVS), the governing body of the veterinary profession in the United Kingdom, stipulates that informed consent is required before treatment may be given to an animal patient. However, we will suggest that more clarity is needed about what exactly this obligation to obtain “informed consent” entails, and what the ethical underpinnings of such an obligation are.^1^ These matters have attracted scant attention in the UK legal literature (Schnobel 2017)^2^—a position which contrasts starkly with the now extensive ethico-legal scholarship analysing the meaning, purpose and ethical underpinnings of the doctrine of informed consent in human healthcare. We begin by examining to what extent, if at all, jurisprudential arguments in favour of recognising patient autonomy, and thus compelling disclosure of the risks of treatment in the human medical context to ensure informed consent, are applicable to veterinary medicine. One recent paper by Ashall et al. (2018) does focus on veterinary informed consent from an ethical perspective, arguing that there is currently an overreliance on owner consent, with the consequence that veterinary professionals may too readily accede to client requests. Having framed informed consent as an ethical pivot point, Ashall et al. conclude that “*what is urgently needed is a thorough re-evaluation of the scope of authority of veterinary informed consent…*” (Ashall et al. 2018:255). Concurring with this conclusion, we propose that such re-evaluation must begin by unpacking competing understandings of decision-making in these professions. In human medicine we trace an incremental shift away from a presumption that the medical professional knows what is best for the patient (the so-called “paternalism” model), towards a presumption that the “reasonable patient” would want to know about the risks and choices inherent in his or her treatment (the “autonomy” model). A strand of case law, culminating in the 2015 Supreme Court decision in *Montgomery v Lanarkshire Health Board,*^3^ confirms that patient autonomy is now the core legal principle underpinning contemporary healthcare law, and that informed consent is the legal mechanism for promoting this autonomy. Corresponding duties have been imposed on healthcare professionals to respect patients’ rights and disclose information. In law, respect for patient autonomy extends to adults who no longer have capacity, with legislation stipulating that reference to previously known wishes should supplement an objective “best interests” calculation.^4^

This current dominance of autonomy has, however, generated criticism in the human medicine context, with commentators arguing that the pendulum has swung too far in favour of autonomy, and that an emphasis on autonomy neglects patient responsibility (Brazier 2006).^5^ In this article we suggest that such concerns apply with still greater force in the veterinary medicine context. The paradigmatic relationship in the human medicine context is the dyadic relationship between health professional and competent adult patient.^6^ The associated assumption is that decision-making is also the product of a transaction between the health professional and the patient. By contrast, in the veterinary context a more complex tripartite relationship exists between the veterinary professional, the owner and the animal patient (McGreevy and Bennett 2010).^7^ Applying the notion of informed consent to this relationship is challenging. Difficulties in ascertaining the wishes of the animal patient are compounded by the animal’s legal status as property or object rather than legal subject.^8^ This seems to entail that the consent to be sought—and therefore the autonomy to be respected—must be that of the owner, rather than the patient. This is reinforced by the fact that the legal agreement will be a contractual one between the veterinary professional and the owner (Mulheron 2017). These factors seem to suggest that in veterinary medicine what matters most is the autonomy of the “client” (or “owner”), who seems to occupy a position analogous to the human “patient”. However, such an assumption is ethically problematic in erasing the interests of the animal, and seems at odds with understandings of companion animal patients as more akin to family members than mere chattels (Fox 2004; Charles and Davies 2008; Ashall and Hobson-West 2017).^9^ In recognition of this, in practice, several provisions in the professional guidance for veterinary surgeons suggest that the owner’s autonomy is tempered by the veterinary surgeon’s professional obligation to provide treatment in the best interests of the animal, and to make animal welfare paramount. For example, on admission to the profession in the UK, veterinary professionals declare an undertaking to “*…ABOVE ALL… ensure the health and welfare of animals committed to my care*” (RCVS 2017b), a statement reinforced by the profession’s Code of Conduct, which states that veterinary surgeons must make animal health and welfare their “*first consideration when attending to animals.*” (RCVS 2017a).^10^

Given these key differences between animal and human medical practice, we argue that caution is needed in importing the language of informed consent and autonomy into the veterinary context and that a new protocol is necessary to inform professional veterinary ethics and practice. Our approach would recognise client autonomy where appropriate, affording it priority in situations where the animal’s welfare will not be compromised by the particular choice of treatment options. However, where welfare concerns exist, we propose a beneficence-based approach to decision-making. This “middle way” recognises the tensions that result from negotiating the veterinarian/owner/animal relationship, which calls into question standard models of patients and health professionals, given both the contested legal status of animals and the way in which veterinary practices effectively operate as businesses involved in delivering private healthcare to human clients who pay for their services. Yet at the core of these interventions both veterinarian and owner have responsibility for the health and welfare of animal patients who can neither indicate their preferred choices, nor refuse proposed treatment. Recognising these blurred boundaries and responsibilities, we suggest that an approach which seeks to integrate animal welfare with owner autonomy not only affords better protection to animal patients, but may go some way to assuaging the significant ethical tensions and associated stress reported by the veterinary profession (Batchelor and McKeegan 2012).

## The Case for Autonomy

### The Rise of Autonomy in Human Healthcare

The concept of autonomy has become prominent over time, to the point where it is now recognised as the dominant principle or value at the heart of health law. The notion of requiring informed consent by the patient was first applied in the context of medical research, following the atrocities committed by Nazi doctors during the Second World War. The resulting Nuremberg Code described this form of consent as follows:

The voluntary consent of the human subject is absolutely essential.

…the person involved should have legal capacity to give consent; should be so situated as to be able to exercise free power of choice, without the intervention of any element of force, fraud, deceit, duress, over-reaching, or other ulterior form of constraint or coercion; and should have sufficient knowledge and comprehension of the elements of the subject matter involved, as to enable him to make an understanding and enlightened decision. (US Government 1949)^11^

Since then, growing awareness of and implementation of human rights (especially post the Human Rights Act 1998), increasing patient access to medical knowledge, and an apparent decline of trust in the medical profession (O’Neill 2002; Case 2017) have led to autonomy assuming ever greater importance in doctor-patient decision-making (Maclean 2009). Before considering the legal implications of this, we clarify how autonomy is understood in the health context.

Mill’s utilitarian version of autonomy has tended to dominate doctor-patient decision-making. Such autonomy, based on self-interest and self-knowledge, can only be overruled to prevent harm to others (Beauchamp and Childress 2013). This version of autonomy can lead to decisions that may seem harmful to outsiders. However, prioritising the autonomy of a competent individual means that even harmful decisions are considered valid. Professional ethical guidelines for doctors promote this Millian version of patient autonomy. For example, in the General Medical Council (GMC)’s 1998 guidance on consent, doctors were advised that:

…you must respect patients’ autonomy - their right to decide whether or not to undergo any medical intervention even where a refusal may result in harm to themselves or in their own death. (GMC 1998)

Explicit reference to patient autonomy disappeared from the revised GMC guidance, issued in 2008, but a similar version of autonomy is implied:

You must respect a patient’s decision to refuse an investigation or treatment, even if you think their decision is wrong or irrational… You must not, however, put pressure on a patient to accept your advice. (GMC 2008)

Therefore, a libertarian autonomy, based on self-interest, is now the dominant version of autonomy enshrined in professional ethical guidance for human patients. The next section considers how this relates to UK health law, which has also witnessed a trend towards the recognition of autonomy, albeit more gradual given the historical tendency of judges to defer to medical expertise in decision-making.

### The Role of Consent in Protecting Patient Autonomy in Human Healthcare

The doctrine of consent plays a key role throughout UK law, as well as in health care provision. If medical treatment is provided in the absence of a valid consent the health professional commits a criminal offence or may be sued in negligence. This has led to UK courts grappling with the question of what information must be disclosed to the patient in order to ensure that his or her consent is legally valid. The 2015 Supreme Court decision in *Montgomery v Lanarkshire Health Board* may be regarded as the culmination of a gradual trend towards recognising autonomy in medical negligence cases. Space precludes a detailed critical examination of the emergence of this complex and contested shift. Rather, here, we outline some of the key moments in the legal history.

Prior to *Montgomery,* a series of cases grappling with the legal obligation to disclose risks were haunted by the spectre of the 1957 decision in *Bolam v Friern Hospital Management Committee.*^12^ In a direction to the jury, later expressly approved by the House of Lords,^13^ McNair J ruled that a doctor was:

… not guilty of negligence if he has acted in accordance with a practice accepted as proper by a responsible body of medical men skilled in that particular art.^14^

This ruling is, of course, a product of an era of judicial deference to medical expertise, and as John Harrington has suggested, it “*can be framed not as wild aberration but as a pragmatic strategy for managing legal contingency”*(Harrington 2017: 18). Moreover, as Harrington demonstrates, under this test for negligence, “*the law opens itself to change in clinical standards as the opinion of ‘responsible practitioners’ shifts over time*.” (Harrington 2017:72). Nevertheless, it took until 1985 for the application of the *Bolam* test to the issue of information disclosure^15^ (as opposed to diagnosis and treatment), to be seriously challenged. *Sidaway* centred on a surgeon’s alleged failure to warn a patient of a less than 1% chance of spinal damage. While the case was unanimously decided in favour of the surgeon, it contained a noteworthy dissenting judgment from Lord Scarman on the appropriate standard to be applied for disclosure of risk, holding that *Bolam* did not apply to the test for risk disclosure (as opposed to diagnosis and treatment). Since there was no question of lack of skill or care in the treatment given by the surgeon involved, Lord Scarman ruled that the question was whether the surgeon:

gave consideration, which the law requires him to give, to the right of the patient to make up her own mind, in the light of the relevant information, whether or not she will accept the treatment which he proposes.^16^

He thereby proposed a “prudent patient” test, as opposed to the *Bolam* doctor-oriented test, thus marking a decisive shift in the jurisprudence on consent and a first step towards incorporation of the doctrine of informed consent into UK law. On the issue of materiality of risk, he suggested the test should be:

…what would a reasonably prudent patient think significant if in the situation of this patient?^17^

Lord Scarman’s reasoning was to prove influential in a series of decisions throughout the 1990s, where a more ‘pro-patient’ view gradually prevailed.^18^ In 1998 this refashioning of *Bolam* was given particular impetus by the House of Lords’ judgment in *Bolitho v. City and Hackney Health Authority,*^19^ which stipulated that in order to be deemed ‘responsible’ a body of medical opinion must be shown to have a logical basis and could not simply be accepted by the court. This ruling signalled the beginning of the end for the dominant position that courts had accorded experts since *Bolam* (Brazier and Miola 2000). It was followed a year later by *Pearce v United Bristol Health Care NHS Trust*^20^ in which the Court of Appeal held that it would usually be necessary to disclose a significant risk which would have affected the judgment of a reasonable patient. Lord Woolf MR noted that:

If there is a significant risk which would affect the judgment of a reasonable patient, then in the normal course it is the responsibility of the doctor to inform the patient of that significant risk, if the information is needed so that the patient can determine for him or herself as to what course he or she would adopt.^21^

As Michael Jones has suggested, Lord Woolf’s dicta appeared to combine a ‘prudent patient’ with a ‘reasonable doctor’ standard (Jones 1999:118), and certainly, as Alasdair McLean notes, there is a significant difference between the *Pearce* test (i.e. that those risks which a reasonable doctor believes the reasonable patient ought to find significant must be disclosed) and a test that would require disclosure of the information that a reasonable patient would want to be told (Maclean 2004). *Pearce* was, however, reaffirmed by the House of Lords in *Chester v Afshar*^22^ which concluded that ‘*medical paternalism no longer rules*.*’*^23^ Although the case ultimately turned on causation, the decision in *Chester* effectively awarded damages to the patient for loss of autonomy, stressing that the court, and not the medical profession was ‘*the final arbiter of what constitutes informed consent’*.^24^ These developments culminated in the 2015 *Montgomery* ruling, when the UK Supreme Court reversed the original ruling of the Inner House of the Court of Session that the plaintiff was not entitled to damages as a result of injuries caused to her child during labour. The Supreme Court upheld the plaintiff’s claim that she should have been warned of the risk of shoulder dystocia and offered a Caesarean delivery. In their speeches, Lords Kerr and Reed considered the main preceding cases, particularly *Sidaway* and its interpretation of *Bolam*, together with the 2008 advice from the General Medical Council, and stated that patients are now legally regarded as:

persons holding rights, rather than as the passive recipients of the care of the medical profession… [and are] … also widely treated as consumers exercising choices.^25^

The reference to patients as “consumers” suggests a Millian approach to autonomy, according to which patients should be provided with all available options and be free to choose whichever treatment they wish, regardless of whether their choice is reasonable. As Harrington observes, the Supreme Court thus draws an explicit distinction “*between a zone in which the profession is largely free to determine technical matters and one in which non*-*technical choices are made by patients and supervised by the courts.*”(Harrington 2017:175). Baroness Hale grounded her understanding of autonomy in bodily integrity, noting that:

the interest which the law of negligence protects is a person’s interest in their own physical and psychiatric integrity, an important feature of which is their autonomy.^26^

*Montgomery,* therefore, upholds the primacy of patient autonomy and can be seen as marking the handing over of responsibility for “*determining the nature and extent of patients’ rights*” from the medical profession to the Courts (Farrell and Brazier 2016:87). However, Farrell and Brazier also acknowledge that for all its symbolic importance, in practice *Montgomery* may have merely aligned the law with current GMC guidance on consent and professional practice.^27^ Others have questioned how radical *Montgomery* is, suggesting, for example, that it would have been more ground-breaking to “*entirely reframe consent in relation to healthcare through the prism of autonomy*-*based human rights….*” (McHale 2017:450). In similar vein, Montgomery and Montgomery have noted the irony that the rhetorical support for autonomy in the judgments sits uneasily with how it disregarded the actual choices of “*an intelligent, educated, articulate, independent and well*-*supported woman*” (Montgomery and Montgomery 2016:89). Nevertheless, the case can be regarded as landmark, partly due to its impact on practice, including the production of revised consent guidelines from the Royal College of Surgeons of England (2016),^28^ and the publication of several articles advising various healthcare professions on consent.^29^

### Critiques of Autonomy in Human Healthcare

Having outlined the rise to prominence of autonomy in the arena of medical-decision making, it is important to stress that lively and ongoing critiques exist, both in practice and in the scholarship. First, commentators have argued that autonomy must be tempered with responsible patient choice, which considers the position of relevant others (Stirrat and Gill 2005). Such views align with those who criticise unfettered autonomy, many of whom prefer a Kantian approach, which is “*not about free choice, but about the drive to appropriate or moral action.*”(Donnelly 2009:19). According to these authors, Kantian autonomy is essentially relational and cannot fail to consider the impact of a decision on others, particularly those in close relationships (Maclean 2009). Similarly, Brazier (2006) claims that the trend in favour of patients’ rights after decades of paternalism has resulted in *over*-*correction* of the imbalance. Pleading for the return of the three “neglected” principles of biomedical ethics: non-maleficence, beneficence and justice—she maintains that, alongside the right to autonomy, patients have reciprocal duties to ensure that their decisions do not breach other principles in terms of the provision of healthcare for others. Thus, responsibility for choice includes considering the consequences of that choice.

Second, autonomy may not suit every human patient in practice. In some circumstances, patients with capacity may wish others to be involved in decision-making, or they may prefer the healthcare professional to make the decision for them, thus abdicating their right to autonomy. For example, with reference to oncological decision-making, Cherny considers that “*relational autonomy*”, which takes into account the patient’s preferences for involvement of her relatives in decision-making, or “*voluntary diminished autonomy*”, where the patient requests that the medical professional makes the decision on her behalf, may be appropriate (Cherny 2012:38).^30^

Third, it is interesting that in *Montgomery* itself Baroness Hale sounds a cautionary note, observing that a patient cannot force her doctors to offer treatment, specifically treatments that are “*futile or inappropriate*.”^31^ Similarly, Heywood and Miola (2017) have highlighted the difficulties even post-*Montgomery* that a patient may face in demonstrating that they have withdrawn their consent. Coggon and Miola (2011) have also contended that courts have tended to confuse autonomy and libertarianism, which can result in untrammelled liberty undermining autonomy in practice, for instance, where the patient does not actually understand the information.

Still more fundamentally, legal theorist Martha Fineman (2004) has contended that autonomy is a flawed concept and that other principles, such as dependency or vulnerability, offer a more appropriate basis for legal decision-making. In summary, compelling arguments exist against the wholesale adoption of autonomy as the sole basis of informed consent in the medical treatment of competent adults. Such arguments apply with greater force in the case of human patients who cannot make decisions for themselves, i.e., adult patients who lack capacity or the not-yet-competent child patient, where proxy decision-makers may be necessary.^32^ We now address whether this claim also holds true for veterinary healthcare, especially given the dependency of companion animals upon their owners.^33^

### The Difficulty of Prioritising Autonomy in a Tripartite Relationship

Perhaps significantly, the UK case law on patient autonomy and the doctrine of informed consent has developed within the context of a straightforward dyadic relationship between health professional and competent adult patient, who are increasingly represented as equal partners in the consent process. As noted above, this may be contrasted with the tripartite relationship existing between the veterinary professional, the owner and the animal patient. As Ashall and Hobson-West (2017:913) observe:

With regards to decision-making in companion animal medicine, a triadic relationship exists between the animal, owner and vet. This makes veterinary medicine ethically complex, especially when the welfare needs of the animal and the wishes of the owner come into conflict. In fact, the tension that veterinarians experience in trying to serve the interests of both animal patients and paying clients has been called the fundamental question in veterinary ethics.

This triadic relationship does not necessarily impact on the *criteria* which must be met for valid consent to be given. As Ashall et al. (2018) have argued, consent in both human and veterinary medicine demand that treatment is freely chosen on the basis of appropriate information disclosure and adequate understanding. However, the *objective* of informed consent is different in each professional setting, since, as Ashall et al. (2018: 255) point out:

Whilst medical consent protects a patient’s rights to make autonomous decisions concerning their own body, veterinary informed consent aims to protect an owner’s right to make autonomous decisions concerning their legal property.

Indeed, the owner can lay claim to consideration as a “consumer exercising choices,” as described by the Supreme Court in *Montgomery.*^34^ Yet, as we argued above, any obligation to respect the owner’s wishes must be constrained by the vet’s paramount professional duty to provide treatment in the best interests of the animal (RCVS 2017a).^35^ These professional obligations echo the legal obligations applicable to children, contained in the common law and the Children Act 1989, which require that child welfare is the paramount consideration.

Of course, analogies between the medical treatment of animals and children are not exact. Children, unlike animals, are deemed to be persons with legal rights and entitlements (Eekelaar 1986; Freeman 1992; Bridgeman and Monk 2000; Stalford et al. 2017), notwithstanding arguments that in many respects they are treated as legal objects.^36^ In addition, no legal mechanism analogous to the wardship jurisdiction, nor any body equivalent to the Official Solicitor’s office (Allen 2017) to protect the interests of animal patients exists. Furthermore, the diversity of animal patients, as regards species,^37^ use, and perceived value to their owners, may influence the ability or willingness of the animal owner to pay for veterinary treatment. And, finally, of course, virtually all veterinary treatment will be delivered as part of a commercial contract, paid for by the owner or their insurance company. This means that legal actions will typically be contractual rather than tortious in nature. However, as Rachel Mulheron (2017) points out, this is unlikely to make a difference in practice, since the scope of the duty owed to the patient under a contractual claim is unlikely to differ from that in the tort of negligence. Rather, as is the case with doctors carrying out procedures under a contract, the contract between the veterinary professional and the owner will have implied into it a duty not to act negligently by exercising reasonable care and skill in diagnosing and treating the condition as well as in advising the owners and disclosing relevant information. However, only in exceptional circumstances will the professional have been taken to guarantee the outcome of the procedure.^38^

Significantly, in the animal context there has not been the discernible shift away from paternalism that we identified above in human medical decision-making. For example, in cases of alleged veterinary negligence or misconduct, the RCVS continues to follow a “reasonable practitioner” standard when advising professionals.^39^ Indeed, the lack of reported cases of veterinary negligence^40^ suggests that there is little prospect that a test for disclosure premised on the “reasonable client” standard will be developed via the courts, as has happened with human patients.^41^

In summary, whilst human medicine has travelled some distance from the “reasonable physician” standard in terms of informed consent, the UK veterinary profession currently seems content to endorse a “reasonable veterinarian” standard. In part this may be due to the obstacles we have identified to applying the concept of autonomy to animals given its grounding in the concept of human rights.^42^ By contrast, animal patients, even those in companion roles, lack legal status and therefore cannot have rights protected by an autonomy-based consent. In short, we consider that the concept of autonomy is neither easily applied to the animal nor to the owner in the veterinary context, and so in the next section turn to consider the merits of an alternative approach.

## The Case for Beneficence

### Beneficence-Based Consent and the Legal Status of Animals

In the UK, animals’ lack of legal status has been reinforced through statute and case law:

Domestic animals, like other personal and movable chattels, are the subject of absolute property. The owner can maintain a claim for their detention or conversion, or for trespass to goods in respect of them, and retains his property in them if they stray or are lost.^43^

Nevertheless, owners face restrictions on how they can treat their ‘animal property’, suggesting that animals comprise a special type of property (Deckha 2012). Thus, the Animal Welfare Act 2006 obliges owners to provide for the welfare needs of their animals, including the treatment of injury and disease. Under section 9.2(e) the owner is responsible for ensuring that an animal is *“protected from pain, suffering, injury and disease.”*^44^ This means that failure to seek appropriate veterinary treatment for an animal could result in the owner being prosecuted under anti-cruelty legislation. However, in contrast to the case of children, there is, as we have seen, no legal mechanism to require owners to comply with veterinary advice through a civil court order.^45^ Moreover, although the RCVS requires veterinary professionals to prioritise welfare, this obligation continues to be balanced against the client’s financial and other considerations. The College advises its members to make decisions about treatment “*based first and foremost on animal health and welfare considerations, but also the needs and circumstances of the client*” (RCVS 2017)^46^ and to “consider the welfare implications of any surgical or other procedure and advise or act appropriately.”^47^ Consequently, notwithstanding certain commonalities in their position as patients, key differences exist between how animal/owner and child/parent relationships are regarded in law. And, although the concept of beneficence or “*doing good for the patient*”(Donnelly 2009:11) is central to decision-making for both types of patient, as we discuss below, much will depend on how this is translated into the legal requirement that best interests be promoted.

Beauchamp and Childress (2013:203) outline two types of beneficence.^48^ “*Positive beneficence,*” also termed “*obligatory beneficence*,” requires agents to provide benefits to others, and may be general (aimed at all others) or specific (aimed at those with whom we have special relationships, such as family, friends, patients). Positive beneficence could be applied to the veterinary professional (for example, an obligation to treat all animal patients, using the best available treatment, regardless of the owner’s circumstances) or to the animal owner (for example, an obligation to fund the best available treatment, even if unable to afford it) but it is difficult to see how this could work in practice, unless limited to “*reasonably necessary treatment*” (Yeates and Main 2010:266).^49^

“*Utility beneficence,*” on the other hand, obliges agents to balance benefits and harms to produce the best results for others. In the case of the animal patient, this would mean balancing the benefits of each treatment option (such as improved quality of life, improved health and the maintenance of the human-animal relationship) with the costs to the client (including financial) and the risks to the patient, to produce the best result in terms of welfare. Utility beneficence recognises that, as veterinary practices are private health enterprises, treatment decisions are often influenced by factors other than the evidence base for available treatments. We now consider how this balance could be achieved in practice, starting with an investigation into the legal form of utility beneficence - the “best interests” standard.

### The Challenge of Calculating Best Interests

For human patients it is clear that “best interests” no longer equates to “best medical interests”(Coggon 2016). Since the decision in *Airedale NHS Trust v Bland,*^50^ the “best interests” standard for incompetent humans has evolved, and primarily as a result of the Mental Capacity Act 2005, it is now necessary to incorporate the patient’s previously stated wishes and values in any decisions where possible (Coggon 2016:409). Best interests can also encompass a patient’s “metaphysical interests”(Coggon 2008:225).

Given current levels of knowledge about animal cognition, it is difficult to ascertain the veterinary patient’s wishes. Superficially, animals can react to any proposed treatment by showing aggression, which could be regarded as a form of ‘dissent’. However, it is only in rare cases that the animal’s preferences are taken into consideration. A more common reaction to such ‘dissent’ is behavioural modification, through training, desensitisation, and the use of food rewards, to persuade such a reluctant animal patient to acquiesce to the treatment decided by the humans involved (Moffat 2008). If the situation is more urgent, then many veterinary professionals advise sedating the patient to enable treatment to be given.^51^ Thus, it is common to prioritise the animal patient’s health interests above other welfare interests.^52^ The situation is somewhat different with child patients, though it is worth noting that there are similar difficulties to the animal patient situation in talking meaningfully about the wishes of a very young child (Alderson 2008). The best interests test originated as a legal requirement in child custody cases at common law, and was later applied in paediatric healthcare (Birchley 2014).^53^ In an ideal situation, parents and healthcare professionals will agree on the best interests of the child. If they fail to agree, then a court declaration should be sought. However, a major problem with an objective “best interests” standard has been the difficulty of defining ‘best interests’. According to Baines (2010),^54^ this may be an ontological problem (there may be no such thing as objective best interests), or it may be an epistemological problem (best interests may exist, but there is no way of discovering what they are). Historically, doctors did tend to consider best interests from a medical perspective (Kennedy 1991), although numerous cases post the Human Rights Act have confirmed that they are now legally required to consider wider interests, and to involve other members of the healthcare team.^55^ For instance, dicta in cases involving sterilisation of incompetent patients, later cited with approval in cases of child patients, have pointed to the breadth of best interest calculations:

When considering the best interests of a patient, it is… the duty of the court to assess the advantages and disadvantages of the various treatment and management options, the viability of each such option and the likely effect each would have on the patient’s best interests and, I would add, his enjoyment of life… any likely benefit of treatment has to be balanced and considered in the light of any additional suffering the treatment option would entail.^56^

Similarly, Section 1(3) of the Children Act offers a list of factors which courts should take into account in determining the best interests of the child, including the risk of any harm, and the importance of assessing the emotional as well as physical needs of the child.^57^ Many high profile paediatric cases involve medical decisions for terminally ill infants, where the interests of the parents and the interests of the child may clash.^58^ Drawing on the dicta cited above in *Re A*, Wall LJ refers to the basis for ‘best interests’ decision-making for children in *Wyatt*:

The judge must decide what is in the child’s best interests. In making that decision, the welfare of the child is paramount. The term “best interests” encompasses medical, emotional, and all other welfare issues (Re A). The court must conduct a balancing exercise in which all the relevant factors are weighed (Re J) and a helpful way of undertaking this exercise is to draw up a balance sheet (Re A).^59^

A similar approach to decision-making for animals could be adopted, though this too is fraught with difficulty, given the animal’s status as property, and the perhaps greater difficulties in ascertaining when animals enjoy life.^60^ As was historically the case with children, no consensus exists on what should be included in a ‘best interests’ calculation for animals. The RCVS Code of Conduct and Supporting Guidance (2017) refers to the “best interests” of the animal on four occasions, without expanding on the meaning of the term. An alternative approach would be to adopt the position that a procedure may be performed provided it is ‘not against’ the animal’s interests (rather than positively in their best interests) as was advocated in some early cases involving children.^61^ However, as Neil Allen has noted, such an approach to treating children is difficult to reconcile either with basic principle or with more recent cases (Allen 2017). This view has recently been confirmed by the ruling in *Alder Hey Children’s NHS Foundation Trust v Evans*. The Supreme Court rejected an argument made by the parents of a terminally ill child that, in evaluating their request to take their child abroad for treatment, the appropriate test should be that such an intervention would not cause ‘significant harm’, even if it could not be regarded as positively in the child’s ‘best interests’.^62^ The Supreme Court was clear that the gold standard for medical decision-making was that the proposed course of treatment was in the child’s interests. This more positive conception of best interests also seems to be accepted by the profession in the case of decision-making about animals. For instance, the British Veterinary Association (2018), in guidance documents on treatment and euthanasia decision-making, promotes a “quality of life” evaluation in conjunction with balancing harms and benefits to both owner and animal, again without further explanation. A useful list of criteria on which to base a ‘best interests’ calculation for companion animals has recently been proposed by Schnobel (2017:253) who suggests that it should include quality of life, the ability to function naturally, the ability to participate in a mutually beneficial companion relationship with the owner, and it should incorporate the veterinary professional’s knowledge of the individual animal and its lifestyle. Arguably, however, the latter criterion depends heavily on the owner’s perceptions and reporting of that lifestyle, in much the same way that ‘best interests’ decision-making for children continues to accord excessive power to parents (Fox and Thomson 2017). We propose that the list should also include an assessment of the risks involved with any proposed treatment, with the veterinary professional best placed to disclose evidence-based information about the level of these risks, and that in line with *Montgomery* this information should be based on what a reasonable client/owner would want to know.

Amongst numerous practical criticisms levelled at the ‘best interests’ test as applied to children, commentators have charged that the standard lacks content, is couched in unclear terminology which offers no meaningful guide to professionals, and that it operates to advance parental and professional interests at the expense of children (Fox and Thomson 2017). Thus, notwithstanding the attempts outlined above to flesh out how the test is to be applied in law in order to encompass a broader range of factors, it remains the case, as Stalford (2017:38) argues, that “*best interests assessments are unnervingly instinctive and highly contingent on the subjective assessment and value framework of the decision*-*maker*”. Indeed, as Heywood’s analysis of the more recent case law demonstrates, it remains extremely rare in practice for parental views to be challenged or overturned in the courts (Heywood 2012). Ultimately, then, parents retain considerable power over decision-making for their children, up to the point where their decisions are deemed to very adversely affect their children’s best interests.^63^ We now reflect on whether animal owners should be endowed with the same authority, and whether it is possible to meaningfully disentangle the interests of animals from their owners.

### Identifying Who Should Protect the Best Interests of the Animal Patient

Perhaps the most striking distinction between decision-making for children and animal patients is the complexity of the relationships and the obligations on the veterinarian in mediating them. Indeed, some authors argue that the veterinary professional has a responsibility to maintain, as far as possible, the unique human-companion animal relationship.^64^ Unfortunately, such a focus on the relationship may reinforce the status of the animal patient as property, and result in a failure to disentangle those interests from those of the owner. For instance, in his critique of the popular UK television programme “Supervet,” Mills argues that treatment is presented as being less important for the animal and more important as a way of fixing a dysfunctional human-animal relationship. “*The animals remain primarily objects, constructed as medicalized bodies requiring fixing in order to assuage the emotional needs of the owner*.” (Mills 2016: 248). This observation lends weight to demands for limits to the scope of owner consent (Ashall et al. 2018). Yet, by highlighting extreme scenarios, it may be argued that authors such as Mills are unduly concerned about clients requesting unethical treatment, given that such situations are likely governed by existing legal sanctions. For example, the potential for causing unnecessary suffering requires both parties to comply with the provisions of relevant animal welfare legislation, although reporting or proving breaches may be problematic given the veterinarian-client relationship.^65^

Is it therefore correct to assume that animal owners are the “best” decision-makers for animals in their charge, echoing assumpions regarding parental decision-making? Building on Salter’s arguments in relation to children,^66^ one could argue that: first, the owner is best placed to report on the animal patient’s preferences and desires and second, it is the owner who will bear some of the burden resulting from any decision, whether this be financial, emotional or relational (Salter 2012). The point about finances is particularly important. As we have seen, veterinary treatment remains an individual contract-based service between the animal owner (client) and the veterinary surgeon (professional). The owner retains autonomy over financial decisions, able to choose between innovative and complicated surgery at one extreme, and euthanasia at the other, and the legitimacy of such decisions being entrusted to the owner is rarely questioned (Mills 2016). Financial constraints on treatment will clearly significantly impact on the owner’s decisions. Thus, any beneficence-based approach must recognise owners’ differing abilities (and willingness) to pay. In addition, when considering companion animals, it could be argued that the owner is in the best position to translate her partiality towards the individual animal (Yeates and Savalescu 2017), into reaching a decision on a “best interests” basis.^67^ Given the need to reconcile these interests, we now turn to framing a proposal for veterinary decision-making which takes account of both the owner’s wishes and circumstances and the veterinary professional’s assessment of the animal’s welfare.

## Conclusion: A Proposal for Equally Weighting Autonomy and Beneficence

To overcome the hypothetical situation where the autonomous and properly informed owner requests treatment for her animal that appears contrary to the animal’s best interests, we propose an alternative approach that provides a compromise between these competing interests. Our approach comprises “*constrained [owner] autonomy*” (Ross 1998:3)^68^ regarding financial commitment, provided that the available options have similar effects on the welfare of the patient, together with *“utility beneficence”* in cases where options with either significant welfare benefits, or negative welfare implications, are under consideration.^69^ At this point, it may be useful to clarify how this “middle way” approach can be deployed in practice, and its implications for consent. In cases where none of the available treatment options have welfare implications for the animal patient (for example, there are several equally appropriate surgical procedures used to repair torn knee ligaments),^70^ there seems no ethical reason why the client’s decision should not prevail. Maximising the client’s financial autonomy means discussing risks and benefits, including post-operative complications and aftercare required, and providing detailed cost estimates for each. It is at this point that the *Montgomery* ruling may have consequences for veterinary practice, if it follows that the level of risk disclosure should approximate that required for human patients. Where welfare differentials exist, a “best interests” discussion can then be usefully employed to prioritise options that promote positive welfare for the patient. This discussion, while taking into account the client’s financial and other constraints, should aim to assist the client in making a decision that maintains positive welfare and, as far as possible, the human-animal relationship, but is achievable for the individual client. In this regard we suggest that useful guidance for veterinary professionals could be derived from the recent more nuanced tests applied to assessing children’s welfare which move beyond the avoidance of physical harm or a purely medical assessment of best interests. For example, in the case of a feline patient diagnosed with hyperthyroidism, there are several possible approaches to treatment. Those involved may take into account the patient’s known dislike for taking oral medication, therefore excluding one potential treatment option, which involves giving oral medication for life (Carney et al. 2016). Such an example suggests how the veterinary profession might derive lessons from the history of informed consent in human medicine, informing and advising the owner to make decisions that promote the best interests of the animal.

In this article we have critically revisited the legal doctrine of informed consent, tracing its rise over the past 30 years in UK law and its conceptual grounding in patient autonomy. Drawing on existing literature, we have outlined key criticisms of the concept, namely that autonomy does not underpin decision-making for every patient, that some patients do not wish to make decisions alone, and that autonomy neglects the interests of others affected by an individual patient’s decision. It is also unhelpful when used as a basis for decision-making by a third party, where other constraints such as finances and welfare apply. Our contribution has been to consider the degree to which this doctrine is transferable to the veterinary context. We have argued that the challenges and tensions in the human field are heightened in the veterinary context, due to the tripartite nature of the animal-client-veterinary professional relationship, and the fact that, even post-*Montgomery,* choice and consumerism dominate the veterinary profession to an even greater extent than its human counterpart.^71^ While it is tempting to assume that veterinary practice can be directly compared with paediatrics, and such an assumption seems to underpin veterinary professional guidance, building on Ashall et al’s (2018) observation that the purposes of consent are different across the two domains, we have argued that a direct comparison is again problematic. Similarly, when evaluating the applicability of a ‘best interests’ approach akin to that used in the paediatric context we have argued that it poses particular problems when it comes to defining the objective best interests of the animal, particularly given the contractual, commercial basis of veterinary treatment which serves to further limit options. Moreover, given the difficulty of ascertaining the animal patient’s wishes, there is inevitably a continuing tendency to prioritise medical over other types of interest. In recognition of these factors, we are supportive of a middle ground which explicitly acknowledges the financial circumstances of the owner. This gives some scope for autonomous decision-making, expressed through freedom of choice between welfare-equivalent options. However, in our view such autonomy must ultimately be constrained where the veterinary professional has concerns about the impact of the client’s decision on animal welfare. As a bottom line, no treatment decision which is against the animal’s interests can be regarded as ethical. Yet we would argue that the ethico-legal obigations on the veterinary professional are more demanding, and, as in the case with children, the gold standard is that treatment decisions should be taken in the animal’s best interests. Here we return to the problem of the absence of specific guidance on the calculation of best interests for animal patients, suggesting that this is an area that would benefit from specific attention by the veterinary profession. In this regard we welcome recent policy moves by the profession to foreground its obligation to promote animal welfare,^72^ and are supportive of calls for more detailed, empirical work to highlight the practicalities of consent and other dilemmas in the veterinary field (Ashall et al. 2018; Hobson-West and Timmons 2015). For example, differing approaches to decision-making may be required for different animal species and the purposes for which they are kept. We accept that major changes to professional regulation, while desirable, are unlikely in the near future, and that for the reasons we have explained there is unlikely to be a body of case law comparable to that which has considered the obligations of health professionals treating humans. However, a key progression would be for the RCVS, as the profession’s regulator, to be more proactive in promoting a “best interests” basis for consent to treatment of animal patients. In short, we would recommend changing the Code of Professional Conduct to explicitly reference the balance that must be achieved between client autonomy and patient best interests when making treatment decisions for animals.^73^

## Footnotes

1 The RCVS recently (March 2018) updated its supporting guidance on informed consent for veterinary professionals, with the main additions being the timing of the consent discussion, who should obtain consent, a list of topics that should be covered during the consent discussion and the purpose of the consent form. It did not include advice on respecting client autonomy. See RCVS 2018, in Supporting Guidance.

2 Schnobel, although not addressing the issue of consent specifically, argues for a “best interests” derived standard of care for animal patients, rather than one based on the commercial relationship between veterinary professional and animal owner.

3 [2015] 1430 AC 1.

4 The Mental Capacity Act 2005 S4.6 states that previous known wishes, beliefs and values should be considered if these can be reasonably ascertained. See also Taylor (2016).

5 Brazier fears that this shift neglects patient duties to avoid harm to others, for example. Interestingly, she paints a bleak picture of a health service based on a ‘consumer-orientated’ model, which reflects the *Montgomery* dicta of ‘patients as consumers’; see also Montgomery (2006).

6 Though this model has been criticised as not reflecting the reality that medicine is delivered by a multi-disciplinary team of health professionals—see, for example, Montgomery (1989) at p332.

7 McGreevy and Bennett describe a companion animal as one used for human companionship, rather than to work or to produce a product. This type of animal patient has been chosen to approximate most closely with the paediatric patient.

8 See, for example, Francione (1995).

9 For example, in Charles and Davies 2008 sociological study of families, a significant proportion of interviewees included pets in their kinship network. Ashall and Hobson-West (2017) have proposed that this kinship be acknowledged in the veterinary context, while Fox (2004) has argued for legal recognition of cross-species kinship.

10 RCVS (2017), at Sect. 2.1.

11 See also Annas and Grodin (1995).

12 [1957] 2 All ER.

13 In *Whitehouse v. Jordan and Another* [1981] 1 WLR 46 as regards diagnosis and treatment and *Sidaway v Board of Governors of the Bethlem Royal Hospital* [1985] 1 AC 1 as regards information disclosure.

14 *Bolam* at 587.

15 See note 13 above.

16 Per Lord Scarman in *Sidaway*, at 876 G-H.

17 Per Lord Scarman in *Sidaway* at 888H; see also Lee (1987).

18 See, for example, *Smith v Tunbridge Wells* [1994] 5 Med LR 334; *Smith v. Salford Health Authority* [1994] Med LR 321 and *Lybert v. Warrington Health Authority* [1996] 7 Med LR 71.

19 [1998] AC 232.

20 [1999] P.I.Q.R. 53.

21 Per Lord Woolf in *Pearce* at 21.

22 *Chester v Afshar* [2005] 1 A.C. 134.

23 Per Lord Steyn in *Chester*, at 16.

24 Ibid, at 14.

25 Per Lords Kerr and Reed in *Montgomery* at 75[G].

26 Per Baroness Hale in *Montgomery* at 108[C]. Counter arguments suggest that autonomy does not equate to bodily integrity, as “*the right to bodily integrity is not lost when autonomy is lost*” for example, through a reduction in mental capacity - Herring and Wall (2017), at 583. How this might apply to animals raises interesting questions, since claims for protection of an animal’s physical integrity may be difficult to sustain, given the interference with bodily integrity permitted by animal welfare legislation, e.g., the Animal Welfare Act 2006 controversially permits the docking of dogs’ tails up to 5 days’ old, provided that they are identified as working dogs (s6(3)) - see Fox (2010). When discussing non-therapeutic sterilisation, even proponents of animal rights have suggested that the right to bodily integrity is less important than the right not to be property, for example, see Fusfeld (2007).

27 See also Heywood (2015); Heywood and Miola (2017); Herring et al. (2017).

28 As an example of its broader implications, in *O’Hare v Coutts & Co* [2016] EWHC 2224 the High Court applied *Montgomery* to a case involving financial advice.

29 See, for example, Bright et al (2017). In this article the authors refer to *Montgomery* as the law on clinical negligence catching up with developments within the professions, as General Dental Council advice pre-*Montgomery* already used a reasonable patient standard.

30 Cherny argues that these types of situation should be respected as “nuanced” forms of autonomy.

31 Per Baroness Hale in *Montgomery*, at 115B.

32 In the case of adult patients, a statutory presumption of capacity exists so that the individual must be presumed to be competent to decide unless proven otherwise according to the statutory test of capacity. Even then, attempts must be made to elicit the patient’s past and present wishes, thus respecting her autonomy. See Mental Capacity Act 2005 and Taylor (n6). It is only then that decisions are made to protect her interests.

33 Satz (2009) has suggested that this dependency renders animals the most vulnerable of all legal subjects - see also Deckha (2015).

34 *Montgomery* at 75H.

35 This duty is emphasised in the Supporting Guidance to the RCVS Code of Professional Conduct, Veterinary Care, which advises that veterinary professionals should *“make decisions on treatment regimes based first and foremost on animal health and welfare considerations….”* at 2.2.c.

36 As Archard and Macleod (2002:1) have suggested, the child is conceived as “if not precisely a thing to be owned… in some sense, an extension of the parent”; see also O’Donovan (1993). Indeed there is some debate as to whether children are owed a legal duty of care by their parents – see dicta by Lady Hale in *Woodland v. Swimming Teachers Association and others* [2014] AC 537, at 589.

37 Although human patients differ in terms of, for example, age, capacity and ethnicity, there is a more obvious “speciesism” in the world of animal treatment. We give different levels of legal protection to different species, and to different members of the same species. Rats can be killed in apparently inhumane ways for pest control, but their deaths are strictly regulated if they are used for research, and there are higher levels of justification required for using certain species of animal, such as primates, dogs and cats, in research. See Animals (Scientific Procedures) Act 1986.

38 See, for example, *Thake v Maurice* [1986] QB 644; see also *ARB v IVF Hammersmith Ltd* [2017] EWHC 2438.

39 See RCVS notes on negligence https://www.rcvs.org.uk/document-library/a-note-on-negligence/ accessed 13 January 2018.

40 A search of legal databases for cases of alleged veterinary negligence in the United Kingdom revealed eight reported cases, of which only two mentioned risk disclosure, and then only as a side issue. See *De Maynard v Streatham Hill Veterinary Surgery* [2001] EWCA Civ 1728 and *Calver v Westwood Veterinary Group* [2001] P.I.Q.R. P11.

41 A sudden surge in veterinary negligence cases is improbable for several reasons. First, there are significantly lower awards available (confined to the economic value of the animal only) for breach of the duty of care to an animal patient, and this is coupled with the costs involved in bringing such a case to court. Recently, the successful introduction of a mediation and arbitration service by the RCVS in 2016 has led to an extension of the trial of the Veterinary Client Mediation Service, leading to even more cases being settled out of court (see https://www.vetmediation.co.uk). Finally, as Fox (2012) notes, it may take a scandal of similar proportions to those that prompted reforms in medical regulation, or a challenge based on human rights legislation, to force change on the profession in terms of how it regulates itself and how it decides cases of alleged misconduct.

42 Heywood (2015:463) illustrates this through the change in judicial language, from “self-determination” to “autonomy”.

43 Halsbury’s Laws of England, Vol 2, Sect. 2.1.6. Absolute Property in Domestic Animals (2017).

44 Animal Welfare Act 2006, S9.2(e).

45 As is the case with the Children Act 1989. The Royal Society for the Prevention of Cruelty to Animals (RSPCA) will, however, often issue improvement notices to owners, rather than prosecuting them, which has some parallels with Specific Orders issued for parents although the RSPCA notices are non-statutory (84,000 improvement notices were issued in 2016, vs. 1400 prosecutions) See https://view.pagetiger.com/RSPCAProsecutionsAnnualreport2016 Accessed 13 January 2018.

46 Supporting Guidance – Veterinary Care at 2.2c.

47 Ibid at 2.2f.

48 We prefer to use the term “beneficence” rather than “paternalism” in situations where the decision-maker is not the patient, thus avoiding the negative connotations of the latter term.

49 Yeates and Main suggest that responsible ownership might include the obligation to fund reasonably necessary treatment. They do not, however, go on to define “reasonably necessary”, but imply that there are limits to the obligation to fund treatment. Veterinary professionals are required, under the RCVS Code of Professional Conduct, to provide immediate first aid and pain relief to any animal, regardless of the owner’s ability to pay (See Supporting Guidance ‘Providing First Aid and Pain Relief’, Sects. 3.7–3.9).

50 [1993] AC 789.

51 See, for example, van Haaften et al. (2017).

52 Indeed, promoting the animal’s health-related welfare is one reason for prioritising human decisions over animal decisions, according to Yeates (2018: 174).

53 Interestingly, in some jurisdictions, there have been moves to incorporate a “best interests of the animal” test in pet custody cases. See Rook (2014) and Deckha (2012).

54 For more on the legal aspects of best interests for children, see Bridgeman (2012).

55 Although Baines (2010) maintains that doctors are actually better placed to decide on a child’s objective best interests than judges, for example.

56 Per Dame Butler Sloss, *Re A (medical treatment: male sterilisation)* [2000] 1 FLR 549.

57 Children Act 1989s.1(3).

58 See, for example, *Portsmouth NHS Trust v Wyatt* [2005] EWCA Civ 1181, where the disagreement between parents and professionals was profound, and the court ruled that the child should be entitled to a “peaceful death”.

59 *Portsmouth NHS Trust v Wyatt* at 87.

60 Although, as more work is published on animal emotions, measurement of “enjoyment of life” may be more achievable. See, for example, Mellor (2011).

61 *SvS* [1972] AC 24 which concerned administration of a blood test.

62 [2018] EWHC 308 (Fam); Supreme Court, Reasons given online, https://www.supremecourt.uk/cases/docs/alfie-evans-reasons-200318.pdf Accessed 8 April 2018.

63 See *Alder Hey Children’s NHS Foundation Trust v Evans*.

64 See, for example, Schnobel (2017), at 254. Also, the development of the term “bond-centred practice” in veterinary literature.

65 As noted earlier, positive duties are placed on companion animal owners and keepers by the Animal Welfare Act 2006, s9.1, making them responsible for ensuring that the animal’s needs are *“met to the extent required by good practice,”* Including protecting the animal from “*pain, suffering, injury and disease.”* However, in cases of non-compliance, vets may be reluctant to report owners to the relevant authorities for reasons of client confidentiality, and clients may be more likely to complain about vets to the RCVS rather than the RSPCA.

66 We have replaced “parent” with “owner”.

67 This might parallel Bridgeman (2012)’s arguments as to why parents should generally be entrusted with making decisions for their children.

68 We have borrowed the phrase from Ross, Chapter 1, replacing “parent” with “owner”.

69 There is a separate argument regarding whether veterinary professionals are, or should be, obliged to offer all available options in every situation, but further exploration of this topic lies outside the scope of this paper.

70 See Aragon and Budsberg (2005).

71 See Montgomery (2006) for a compelling critique of health law’s endorsement of consumerist rhetoric.

72 See Vet Futures ‘Taking charge of our future: A vision for the veterinary profession for 2030’ (RCVS/BVA 2015) Section 4.5.4 “Clarify and promote regulatory requirements and professional responsibilities relating to animal welfare” p21 https://www.vetfutures.org.uk/resource/vet-futures-report/ Accessed 13 February 2018. Arguably, since the veterinary nursing profession has always been vocal regarding its responsibilities for animal welfare, this move may have been influenced by its sister profession.

73 The RCVS could, for example, follow the lead of the British Veterinary Association in ‘Vets Speaking Up for Animal Welfare: BVA Animal Welfare Strategy’ (2016, p20) which states that “Promoting a patient’s best interests sometimes requires ethically appropriate influencing of animal owners.”.
